# Effects of Shiitake (*Lentinus edodes* P.) Mushroom Powder and Sodium Tripolyphosphate on Texture and Flavor of Pork Patties

**DOI:** 10.3390/foods9050611

**Published:** 2020-05-10

**Authors:** Soonsil Chun, Edgar Chambers, Delores H. Chambers

**Affiliations:** 1 Department of Food and Nutrition, Sunchon National University, Jeonnam 57922, Korea; css@sunchon.ac.kr; 2 Sensory Analysis and Consumer Behavior, Kansas State University, Manhattan, KS 66506, USA; delores@ksu.edu

**Keywords:** pork, descriptive, sensory, shiitake mushroom, flavor, texture, phosphate

## Abstract

Increasing consumer desire for functional food ingredients, including such PRODUCTS as shiitake mushroom (*Lentinus edodes* P.) powder (SM), demands that the sensory impact of such ingredients be tested in an appropriate food system. Pork patties are a common food in many Asian countries. Pork patties in this study were prepared with and without SM, an ingredient that is gaining popularity around the world. A lexicon for describing the texture and flavor of cooked pork patties, with and without 0.5% sodium tripolyphosphate (STP), a typical additive to meat, and with varying amounts of SM (0% to 6%) was developed by a highly trained panel to compare sensory properties for each type of patty. The attributes evaluated were juiciness, toughness, rubberiness, mealiness, pork identity (pork ID), meatiness, mushroom, onion, garlic, black pepper, heat/burn, soapy, chemical, animal hair, fatty, salty, sour, bitter, slick, and astringent. An addition of 0.5% STP produced more intense ratings for soapy, salty, sour, and astringent attributes. Without STP, patties containing shiitake mushroom powder had a more mealy consistency but more pork ID than they did with STP.

## 1. Introduction

Flavor is one of the most important factors contributing to consumers’ perception of the quality of meat products [1,2]. The flavor of pork has not received as much attention as beef and chicken in recent years, although it is one of the most frequently consumed meats worldwide. Raw pork has little odor and only a mild, serum-like taste, which is described as salty, metallic, and bloody-tasting, with a sweet aroma [3,4]. During the cooking process, numerous nonvolatile compounds in meat, including pork, undergo degradation, producing hundreds of volatile compounds that together make up the characteristic “pork” flavor [5]. Similarly, mushrooms can impart flavor properties into the products they are added to. For example, the shiitake mushroom (SM) has a unique flavor compound (lenthionine) [6] and also contains low to moderate levels of umami flavor potentiators, such as 5′ribonucleotides [7]. The number of nucleotides and other compounds tested and the impact on the savory character commonly associated with the umami taste when mushroom is blended into meat depends on how the mushrooms are processed. Higher levels of heating during mushroom processing has been found to give a more savory flavor to beef patties [8].

Texture is another attribute that consumers notice when judging the quality of meat. European studies of consumers’ perceptions of “quality” and “wholesomeness” of the pork they purchase, compared with the same attributes judged after consumption of the pork, showed that consumers tended to prefer paler, softer meat when making purchases, even though such attributes had little relationship to their enjoyment of the pork once it was consumed [1,5,9,10].

Shiitake mushrooms (SM) are nutrient-dense. In Asia, these mushrooms are thought to have special medicinal properties with respect to several diseases, including diabetes, anemia, various forms of cancer, and oral health [11,12,13,14,15,16]. The way in which the mushrooms are prepared can play a role in the nutrient retention. Lee and others [17] found that microwaving and roasting the mushrooms caused the most vitamin and mineral retention. Other authors [18] found that the stipes and caps of shiitake mushroom have different nutritional value, but the same flavor. Several authors have studied the consumer acceptability of meat products with added mushroom powder and found that the products were acceptable [19,20]. The use of adjuncts in meat products is becoming more popular as consumers are looking for healthier alternatives to the traditional patty. For example, Taylor et al. [21] investigated the addition of tempeh (a fermented soybean product) into beef patties at varying amounts and found that it affected sensory attributes. Phosphates are known to increase water-binding and stabilize meat emulsions, improve juiciness and tenderness, provide mineral supplementation, and maintain the flavor of processed meat products [22,23,24]. Phosphate in pork and beef roasts allowed them to be reheated after 1 and 3 days of refrigeration with minimal loss of juiciness, tenderness, or flavor intensity [25]. Phosphates have been shown to increase acceptance of turkey patties [26], but the impact of phosphates on flavor is somewhat dependent on differing human sensitivity to phosphate compounds [27].

Various authors [28,29,30,31] have pointed out the importance of studying sensory properties of products, including meat products when adding new ingredients, such as functional ingredients, to existing products. The flavor properties of many foods have been well-described, defined, and referenced. For example, Maughan and Martini [32] found that fresh cooked pork is characterized by such terms as brothy, fatty, salty, sweet, and umami. Other authors [33] showed that consumer acceptance of cooked, stored pork patties increased after the addition of plant extracts containing antioxidants. Storage, particularly at refrigerated temperatures, can result in a warmed-over flavor (WOF) when products are reheated. This occurs largely because of compounds produced from lipid oxidation [34]. Controlling WOF, especially with natural antioxidants, is important because of the increase in precooked meat products [35,36]. Various authors have found that mushroom extracts contain antioxidants [37,38], which could potentially reduce WOF in stored pork patties. The mushroom flavor could also mask some off flavors such as WOF, although the mushroom flavor is greatly reduced during drying [39].

The objectives of this study were to a) determine flavor attributes of pork patties when mushroom were added, b) compare the flavors of fresh, cooked pork patties with and without 0.5% Sodium tripolyphosphate (STP) and with either 0%, 2%, 4%, or 6% shiitake mushroom powder (SM), and c) determine the impact of refrigerated storage on cooked pork patties with and without those same levels of compounds.

## 2. Materials and Methods

### 2.1. Raw Sample Preparation

Ground pork was purchased frozen from a local food market in Manhattan, KS (Dillon’s, distributed by Kroger Co, Cincinnati, OH 45202), thawed in the refrigerator, and processed as patties within 12 h of purchase. The meat (800 g) for each batch of patties was mixed with iodized salt (Kroger), black pepper (McCormick), garlic powder (McCormick), and water (48 mL). The recipe was determined based on pre-testing various recipes found online and in cookbooks; the final ingredients and amounts were chosen from among those commonly used in recipes without adding other competing ingredients found in some recipes (e.g., coriander, carrot). The appropriate amount of STP (0% or 0.5%) and shiitake mushroom powder (Jangheung, Korea; 0% to 6%) were added (Table 1) and mixed thoroughly using a KitchenAid mixer (model Pro600, Benton Harbor, MI) with a paddle attachment on a medium setting for 30 s. Ten patties were prepared with 80 g of meat mixture using a 9 cm Tupperware^®^ (Orlando, FL) patty maker, resulting in a patty 1 cm thick. Patties from each experimental batch were frozen at −20 °C prior to their use in the sensory tests.

### 2.2. Cooked Sample Preparation

Before cooking, patties were thawed overnight in a refrigerator at 4 °C. Each 80 g pork patty was cooked in a 204 °C convection oven until the internal meat temperature reached 74 °C (approximately 16 min). Trays of patties were rotated half-way through the cooking process to help ensure that heating was consistent among the patties. For stored samples, cooked pork patties with and without STP and/or SM were wrapped in aluminum foil and stored for 48 or 96 h at 4 °C in a commercial refrigerator. For serving, patties were unwrapped and reheated in a microwave oven with a rotating tray before testing by the panel.

### 2.3. Panelists

Five professionally trained panelists (all female) from the Center for Sensory Analysis and Consumer Behavior, Kansas State University, Manhattan, KS comprised the descriptive sensory panel for this study. The panelists had completed 120 h of training on a broad range of products in all aspects of descriptive sensory techniques, including attribute identification and scaling. Panelists also had more than 1000 h of experience in general sensory testing for a wide variety of foods, including meat products. Panelists were required to participate in periodic revalidation and retraining during their tenure in sensory analysis testing. Such numbers of highly trained panelists have been shown to be able to discriminate among samples better than larger panels of less trained panelists [40,41,42]. Similar panels have been used for other recent studies [43,44,45].

### 2.4. Sample Serving

Patties were cut crosswise into four equal pieces and served on a 6 inch foam plate resting on a warmed brick, to keep the samples at the proper temperature. All samples were assigned three-digit codes, and separate codes were used for lexicon development and actual testing. Eight pork patties, control and with combinations of SM and STP, both fresh and stored were examined during the orientation sessions to develop a lexicon (terminology, definitions, and references) for describing the texture and flavor characteristics. For actual testing, eight sessions for each storage period were held in random order for sample evaluation. During one session, a sample, labeled with a three-digit random number, was served to the panel. After 20 min, another sample of the same product variation was provided to the panel to ensure that samples were evaluated while warm. This re-serving of the samples was continued until the panel had completed their consensus evaluation (three samples of the same variation were usually served in a session, but occasionally only two, or up to four samples were needed). Individual panelists evaluated the intensity of each attribute using the lexicon and ballot developed in the orientation sessions. Intensities were scored on a 0- (none) to 15- (extremely strong) point scale divided into half-point increments. Scores lower than 5 were considered low, scores of 5 to 10 were considered moderate, and scores higher than 10 were considered high in intensity on this scale. Deionized water, unsalted crackers, carrots, and Lipton^®^ tea were provided to cleanse the palate during testing. Hot Lipton^®^ tea was prepared according to the directions on the package and was used because the warm liquid and flavor of the tea appeared to remove and neutralize the fatty, soapy flavor sometimes remaining in the mouth between samples.

### 2.5. Lexicon Development

The lexicon development method is a generalized consensus method [46] adapted from the profile method of flavor analysis [47]. Vocabulary differences were discussed, and agreement was reached on attributes, definitions, and references. The use of consensus for vocabulary development is important to ensure that each panelist understands and can agree on the terminology used, as well as the intensities associated with each reference for the attribute.

### 2.6. Sample Evaluation

For testing, samples were cooked and stored, if appropriate, and served to the panelists, one sample variable per session. Panelists were not told whether they were evaluating any of the same samples used in orientation. The same consensus approach was used as during the orientation and lexicon development phase. Consensus evaluation during testing is an effective way to ensure that key attributes, such as storage attributes or ones that have not be apparent in samples during lexicon development, can easily be added and scored consistently by the panelists [46] and has been used effectively for various products in published studies for many years [48,49,50,51,52,53,54]. When the panelists reached consensus on the attributes and intensities noted in that sample, testing was completed for that sample. The profile for the fresh control sample with no added phosphate was determined first (requiring four 1 h sessions) by the panel. Other samples were served on successive days in random order, and their profiles determined.

### 2.7. Data Analysis

Statistical analysis by ANOVA or other methods used to differentiate “means” is inappropriate for flavor profile data because all panelists consent to a single-intensity value (consensus value), resulting in no variance [46]. By definition, differences in scores, which have no variance because they are consensus scores, are different from each other. In practicality, many users of consensus methods use a difference of 1 point as showing an important difference between two samples.

The data from consensus profiles can be, and was used in a Principle Components Analysis (PCA) in SAS^®^ version 9 (SAS Institute, Cary, NC, USA) to “map” the products and attributes for comparison.

## 3. Results and Discussion

### 3.1. Lexicon

The final lexicon for describing texture and flavor characteristics of pork patties is provided in Table 2. All 20 attributes were used to identify characteristics in several variations of patties. The terms are similar to those used by other authors for pork [3,4,5] and for samples with added phosphates [25,27], although the terminology used in this study tends to be more detailed.

### 3.2. Fresh Patties: Texture and Flavor Results

Intensities for the texture and flavor, including mouthfeel attributes (Table 3), show that differences were obtained by the panel, differentiating among cooked pork patties with or without 0.5% STP and with either 0%, 2%, 4%, or 6% SM.

Intensities for the texture and flavor, including mouthfeel attributes (Table 3), show considerable differences in samples for many attributes. For texture, samples containing SM and no STP generally had higher juiciness, were less rubbery, and were mealier than samples with STP. Regardless of the amount of SM added, the addition of STP resulted in samples without any mealy character. Juiciness appeared to decline in samples with both STP and SM added at higher amounts, suggesting that the combination of the two ingredients either bind moisture so much that it is not released when chewed, or that those samples had a high level of cooking loss. Unfortunately, neither of those physical measures were taken in this study. Other researchers have shown that STP added to turkey patties increased consumer perception of juiciness and decreased firmness.

Examination of flavor profiles for the different types of pork patties showed that pork patties without STP were rated higher for pork ID, meatiness, onion, garlic, pepper, and fatty character. Samples containing 0.5% STP generally had higher ratings for soapy, salty, and sour attributes. The soapy attribute, which has been noted by other researchers [22,25,27], was at a low intensity, but showed a trend of increasing intensity in STP containing samples when higher levels of SM were added. Soapiness is often considered an undesirable flavor characteristic in pork patties, and appeared to be related to the presence of STP. However, prior research has shown that off-flavors from STP are inconsistently perceived by consumers [26] in chicken patties. The addition of STP increased acceptance of pork patties regardless of addition of SM for Korean consumers, and increased acceptance among US consumers, but only when SM was not present or at low levels [20]. In addition, recent research [55,56,57] has shown that consumers want more “natural” foods, and the use of ingredients with “chemical-sounding names” (in this case, sodium tripolyphosphate) result in reduced perceptions of naturalness. Morse [58] reported an unacceptable soapy flavor when STP was used in meat. A mouthfeel designated as “soapy” was found in STP-containing samples, and might be considered an off-flavor. Hargett and others [59] found that an increase in the sourness of frankfurters was correlated with the use of sodium acid pyrophosphate. An astringent attribute was noted to be an important contributor to the overall flavor of pork. This astringent attribute, as well as the basic tastes, has too been found in the evaluation of other muscle foods [60,61].

The addition of SM powder alone slightly decreased pork ID and increased mushroom character, although the meaty flavor remained at a moderate level unless 6% SM was added, at which point the samples had less meaty flavor than the control without STP or SM. The addition of 6% SM also increased the chemical flavor regardless of whether STP was added. The addition of either SM or STP seemed to mute the intensity of other additive flavors, such as onion, garlic, and pepper. High levels of SM (6%) combined with 0.5% STP produced the highest intensity ratings for the attributes soapy, chemical, bitter, and animal hair, suggesting that 6% SM is too high a percentage for addition to pork patties. Other research has shown that addition of 6% SM with STP was disliked by US consumers, although it was liked by Korean consumers [20].

### 3.3. Stored Patties: Texture and Flavor Results

Sensory data for patties that had been stored for 48 h or 96 h after cooking are presented in Table 4 and Table 5, respectively. Storage of cooked pork patties for 48 h and 96 h, with STP, increased the perception of astringency (Table 4 and Table 5) compared to fresh samples. Storage for 48 h led to increased intensity ratings for soapiness and bitterness, and reduced intensity ratings for fat, pork ID, meatiness, and brown/roasted flavors (Table 4). Addition of SM alone and subsequent 48 h storage produced increases in sourness, meatiness, protein, and brown/roasted attributes, but reductions in pork ID. Addition of both SM and STP followed by 48 h storage yielded increases in salty, meaty, and brown/roasted flavors and reductions in protein and bitter flavors. Storage of patties, regardless of addition of SM or STP, decreased juiciness, and produced a warmed-over flavor (WOF), an unspecified protein note, and a brown/roasted character that might be associated with Maillard browning occurring during the reheating process in samples with less moisture.

After 96 h of storage, patties with SM alone had reduced intensities for most attributes, with the exception of fat perception and an increase in WOF. Use of both SM and STP, followed by 96 h storage, produced increases in ratings for salty, sour, brown/roasted, and pork ID; only occasional decreases in ratings were noted at this storage time compared to 48 h.

### 3.4. Principal Component Maps (Biplots) of Sensory Data and Pork Patty Variations

**Figure 1:**
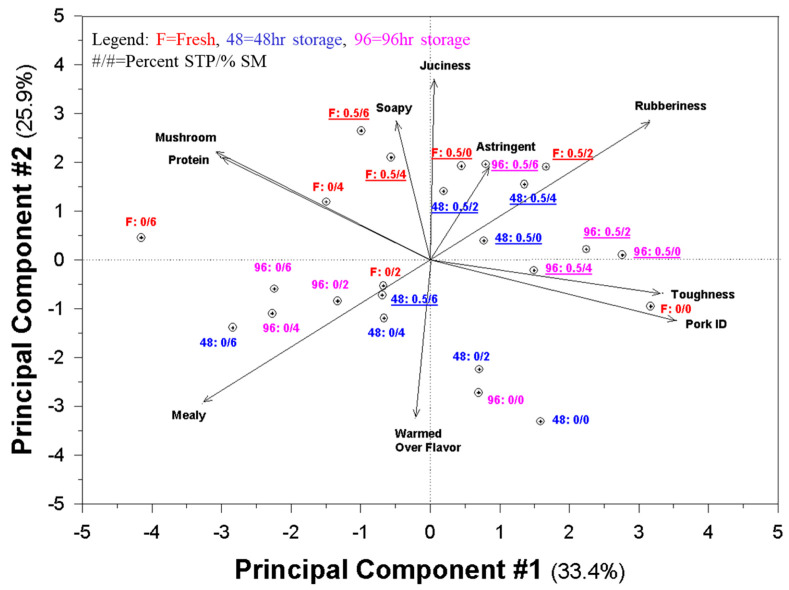
Principal component analysis (PCA) plot relating samples varying in amount of shitake mushroom powder and sodium tripolyphosphate to attribute intensities given by the descriptive panel.

**Figure 2:**
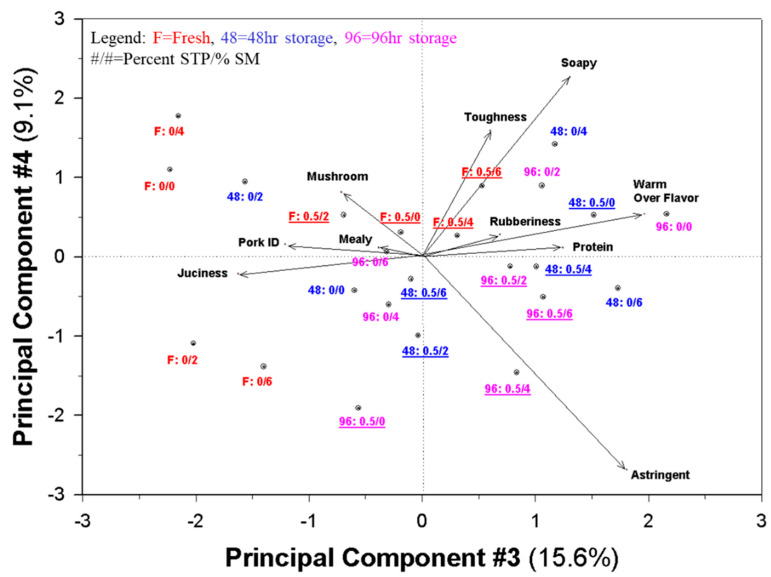
Principal component analysis (PCA) plot relating samples varying in amount of shitake mushroom powder and sodium tripolyphosphate to attribute intensities given by the descriptive panel.

The sensory maps (Figure 1 and Figure 2) clearly show the impact of the addition of SM and STP, as well as age on the samples. Although it is typical to show only the first and second principal components (PCs), the third and fourth PCs account for approximately 25 percent of the variation. Other authors have noted that using only a single map showing two dimensions can be problematic because the mapping procedure is based on overall data and can distort interpretation without careful review of other data sources, including additional PCs and the original data [62,63].

In this case, in Figure 1 it can clearly be noted that the addition of SM increases mushroom, protein, and meaty flavors and reduces the specific pork ID character in the patties. Addition of STP increased rubberiness and astringency. Toughness, pork ID, and juiciness also tended to increase with the addition of STP. However, Figure 2 shows that the increase in the general protein flavor is dependent on storage; fresh samples did not show that character.

Both Figure 1 and Figure 2 show the interaction of the addition of SM, STP, and time. All samples of any one type tend to be scattered into at least two quadrants of the maps, which suggests that the one variable is dependent on the level of at least one other variable. However, the maps still show a rather cohesive picture that differentiates fresh from stored samples, samples with and without SM, and those with and without STP.

One obvious limitation of this study is that no consumer data was collected on stored samples, although prior consumer studies were published on similar fresh samples [20]. However, no comparison can be made between this study and prior consumer studies because of the complexity of the consumer response and limitations on the number of samples.

## 4. Conclusions

In this study, 19 flavor and texture attributes (four textures, 13 flavors, and two mouthfeel attributes) were identified in pork patties with added SM and STP. Those attributes include ones related to fresh pork flavor, stored pork flavor, and the characteristics imparted by STP and SM when added to pork. Specific lexicons are valuable for use by future researchers to understand additional issues associated with such ingredients in products.

Shiitake mushroom powder alone (i.e., without added phosphate) increased the juiciness and decreased the toughness and rubberiness of the pork. There was no change in pork ID with added SM, although overall meatiness decreased with a concomitant increase in mushroom flavor. Mushroom flavor could include the impact of the umami character in mushrooms. SM also slightly increased the soapy character of the patties.

As expected, pork patties with added STP alone were less tough and had much more rubberiness than those without STP or SM added. STP also decreased pork ID, meatiness, and increased soapy character and astringency.

Increases in juiciness, decreases in toughness and rubberiness, along with higher levels of pork ID, meatiness, and a lack of off-notes, such as soapy and astringency, are generally positive things for meat products. The impact of mushroom flavor notes is dependent on culture [20].

As expected, storage did not result in any potentially positive changes in pork patties, although it must be stated again that consumer studies were not conducted on stored samples and consumers could respond differently than expectations. Storage for 48 or 96 h decreased juiciness regardless of whether SM, STP, or both were added. Mealiness tended to increase over time with or without SM, but did not develop in those products containing STP. Many flavor attributes, such as the spice/seasoning notes and basic tastes, changed little over time regardless of additives, suggesting that those flavors are unaffected by the storage process. In addition, there was little change in samples which were fresh or had been in 48 h of storage, with the exception that WOF appeared as expected. Unfortunately, neither SM nor STP appeared to reduce WOF. At 96 h of storage, pork ID and meatiness tended to slightly decrease with added SM, and pork ID slightly increased with STP. In addition, a rancid note appeared in one sample containing STP. Astringency was increased slightly at 96 h with added STP, and this was not affected when SM powder was added to those samples.

Overall, SM and STP impacted both the flavor and texture of the pork patties and did little to mitigate storage effects. The presence of SM increased the perception of mealiness in pork patties and did not seem to consistently enhance the protection from cooking losses and development of WOF gained by the addition of STP. Although STP may contribute to undesirable flavors when added to pork patties, advantages in improved texture and overall acceptability can be gained by the addition of this chemical [64]. The data show that these additives were generally not effective in reducing aspects of stored pork that are considered undesirable (e.g., WOF, rancidity, astringency) by many researchers. Future studies should evaluate individual attributes of pork patties with additives, and how the changes in specific sensory characteristics are different. Understanding specific characteristics will allow researchers to better understand the impacts on consumer acceptance.

## Figures and Tables

**Table 1:** Formulation of experimental batches.

Phosphate (%)	Shiitake (%)	Pork (g)	Onion (g)	Salt (g)	Black Pepper (g)	Garlic Powder (g)	Water (mL)	Phosphate (g)	Shiitake Powder (g)
0%	0%	800	80	8	1.6	0.8	48	0	0
0%	2%	800	80	8	1.6	0.8	48	0	16
0%	4%	800	80	8	1.6	0.8	48	0	32
0%	6%	800	80	8	1.6	0.8	48	0	48
0.5%	0%	800	80	8	1.6	0.8	48	4	0
0.5%	2%	800	80	8	1.6	0.8	48	4	16
0.5%	4%	800	80	8	1.6	0.8	48	4	32
0.5%	6%	800	80	8	1.6	0.8	48	4	48

**Table 2:** Terms, definitions, references, and intensities for describing texture and flavor characteristics of pork patties.

Attribute	Definition	Reference ^a^ and Intensity ^b^
**Texture**		
Juiciness	The amount of liquid expressed from the sample during the fourth chew.	Hormel Cure 81 Ham = 5.0
Toughness	Degree of force required breaking through the surface of the sample with the molars. (Low to high)	Hormel Cure 81 Ham = 9.0
Rubbery	The perception by which individual meat pieces bounce away from molars during mastication.	Oscar Mayer Fat Free Wiener = 7.5
Mealy	The perception of fine, soft particles distributed within the product.	Beef Liver = 10.0
**Flavor**		
Pork ID	Meat aromatics that can be specifically identified as cooked pork.	Pork Patty = 9.0 (flavor)
Meatiness	The perception of aromatics associated with meat products.	Pork Patty = 10.0 (flavor)
Mushroom	Aromatics associated with dried shitake mushrooms such as brown, musty/earthy, and dried mustard-like.	Shitake Mushroom Powder in water = 7.5 (flavor)
Onion	The aromatics commonly associated with onion characterized as sweet and slightly pungent.	Chopped Fresh White Onion = 7.0 (flavor)
		McCormick Onion Powder = 9.0 (aroma)
Garlic	Aromatics associated with garlic.	McCormick Garlic Powder = 9.5 (aroma)
Black Pepper	Spicy, pungent, musty and woody aromatics characteristic of ground black pepper.	McCormick Ground Black Pepper = 13.0 (aroma)
Soapy	An aromatic associated with hand soap.	0.25% solution of Ivory bar soap = 2.0
Chemical	Aromatic associated with garden hose, hot Teflon pan, plastic packaging.	Lightly burnt (Singed) plastic in covered beaker = 13.0
Animal Hair	The aromatic perceived when raw wool is saturated with water (not including soap, solvent, or lanolin aromatics).	Wool in water = 9.5 (aroma)
Fat	Flavor associated with fat. Ranging from light to heavy.	Wesson Vegetable Oil = 7.0 (aroma)
		Pork Patty = 7.5 (flavor)
		Wesson Vegetable Oil = 10.0 (flavor)
Salty	A fundamental taste sensation of which sodium chloride in water is typical.	0.15% Sodium Chloride Solution = 1.5
		0.2% Sodium Chloride Solution = 2.5
		0.25% Sodium Chloride Solution = 3.5
		0.35% Sodium Chloride Solution = 5.0
Sour	A fundamental taste sensation of which citric acid in water is typical.	0.015% citric acid solution = 1.5
		0.025% citric acid solution = 2.5
Bitter	The fundamental taste sensation of which caffeine in water is typical.	0.01% caffeine solution = 2.0
		0.02% caffeine solution = 3.5
		0.035% caffeine solution = 5.0
**Mouthfeel**		
Slick	A feeling factor (possibly textural) that gives the impression of smoothing the surfaces of the mouth, especially the tongue.	Quaker Quick Oats Oatmeal = 12.0
Astringent	The chemical feeling factor on the tongue or oral cavity that can be described as puckering or dry.	0.03% Alum Solution = 1.5
		0.05% Alum Solution = 2.5

^a^ Generally, references were served at room temperature; ^b^ Intensities based on a 0 (none) to 15 (extremely strong) scale.

**Table 3:** Flavor and texture profile scores for pork patties with varied percentages of shiitake mushroom powder with and without phosphate.

	Sodium Tripolyphosphate (STP)							
	0%				0.50%			
Shiitake	0%	2%	4%	6%	0%	2%	4%	6%
**Texture**								
Juiciness	6.5	7.0	8.0	7.5	7.0	8.0	6.0	6.5
Toughness	9.0	5.0	6.0	3.5	6.0	8.0	5.5	6.5
Rubbery	5.0	2.0	4.0	0.0	6.5	6.0	5.5	5.0
Mealy	0.0	3.0	3.0	4.0	0.0	0.0	0.0	0.0
**Flavor**								
Pork ID	7.0	5.5	5.0	3.0	4.5	5.5	4.0	3.5
Meatiness	9.0	8.0	8.0	4.5	7.5	9.0	8.0	6.5
Mushroom	0.0	2.0	5.0	4.0	2.0	2.5	4.0	5.0
Onion	5.0	3.0	3.5	2.5	2.0	2.5	2.0	1.0
Garlic	2.5	1.5	2.5	0.0	1.0	2.0	1.5	1.0
Black Pepper	3.5	2.5	4.0	2.0	2.0	3.5	2.5	3.0
Heat Burn	0.0	1.5	1.5	0.0	1.5	2.0	0.0	1.5
Soapy	0.0	0.0	2.0	0.0	2.0	2.0	2.5	3.0
Chemical	0.0	2.0	0.0	2.0	0.0	0.0	0.0	4.0
Fat	5.0	5.0	4.0	4.5	4.0	4.0	4.0	4.0
Salty	3.0	3.0	2.5	3.0	3.0	3.5	3.0	3.5
Sour	1.5	1.5	1.5	2.0	2.5	2.0	2.0	2.5
Bitter	2.0	2.0	2.0	2.0	2.0	2.0	2.0	3.0
**Mouthfeel**								
Slick	0.0	0.0	1.5	2.0	0.0	0.0	0.0	0.0
Astringent	0.0	1.5	0.0	1.5	1.5	2.0	2.0	2.0

**Table 4:** Flavor and texture profile scores for pork patties with varied percentages of shiitake mushroom powder with and without phosphate, and with 48 h storage.

	Sodium Tripolyphosphate (STP)							
	0%				0.50%			
Shiitake	0%	2%	4%	6%	0%	2%	4%	6%
**Texture**								
Juiciness	4.0	5.0	4.5	4.5	6.0	6.0	6.0	5.5
Toughness	7.0	7.0	6.5	5.0	6.5	5.5	7.0	6.0
Rubbery	2.0	3.5	2.5	2.0	6.0	5.5	6.0	5.0
Mealy	2.5	2.5	2.5	5.0	0.0	0.0	0.0	2.0
**Flavor**								
Pork ID	6.5	6.0	5.0	3.0	5.0	4.5	5.5	4.5
Meatiness	9.0	9.0	8.0	6.0	7.0	8.5	9.0	7.5
Mushroom	0.0	2.0	3.0	1.5	0.0	3.0	3.5	3.5
Onion	2.5	2.5	2.5	3.0	2.5	2.0	2.0	2.0
Garlic	1.5	2.0	2.0	1.5	1.5	1.5	1.5	1.5
Black Pepper	3.0	2.0	3.5	3.0	3.0	2.5	3.0	1.5
Heat Burn	1.0	0.0	2.0	1.5	0.0	0.0	2.0	0.0
Soapy	0.0	0.0	3.0	1.5	0	1.0	2.5	2.0
Chemical	0.0	0.0	0.0	0.0	0.0	0.0	2.0	2.0
Fat	4.0	3.5	3.5	3.5	3.5	3.5	4.0	4.0
Protein	0.0	1.0	2.0	4.0	4.0	2.5	2.5	2.5
Brown/Roasted	5.5	4.5	5.0	5.0	4.0	4.5	4.5	4.5
Warmed-Over Flavor	2.0	1.5	3.0	2.5	1.5	1.5	1.5	1.5
Salty	3.0	3.0	3.0	3.5	3.0	3.5	3.5	3.5
Sour	2.0	2.0	2.5	2.0	2.0	2.0	2.0	2.5
Bitter	2.0	1.5	2.0	2.0	2.5	2.0	2.0	2.0
**Mouthfeel**								
Slick	0.0	0.0	0.0	0.0	0.0	0.0	1.0	0.0
Astringent	1.5	0.0	1.5	2.0	1.5	2.5	3.0	1.5

**Table 5:** Flavor and texture profile scores for pork patties with varied percentages of shiitake mushroom powder with and without phosphate, and with 96 h storage.

	Sodium Tripolyphosphate (STP)							
	0%				0.50%			
Shiitake	0%	2%	4%	6%	0%	2%	4%	6%
**Texture**								
Juiciness	3.0	5.0	5.0	6.5	6.0	7.0	6.0	6.5
Toughness	9.5	6.0	5.0	5.0	6.5	7.0	6.5	8.0
Rubberiness	4.0	2.5	2.0	1.0	6.0	5.5	5.5	6.0
Mealy	2.5	3.0	3.5	4.0	0.0	0.0	0.0	0.0
**Flavor**								
Pork ID	4.0	4.5	4.0	4.5	5.5	6.5	5.5	3.5
Meatiness	7.5	7.5	5.5	5.5	9.0	8.0	9.0	7.0
Mushroom	0.0	3.5	4.0	3.5	0.0	0.0	1.5	2.0
Onion	3.0	25	2.5	2.0	3.0	2.0	3.5	2.0
Garlic	2.5	1.5	2.0	1.5	2.5	1.5	1.5	1.5
Black Pepper	3.0	3.0	2.5	2.0	2.0	2.0	2.0	1.5
Heat Burn	0.0	1.5	0.0	0.0	1.0	0.0	0.0	0.0
Soapy	0.0	2.5	2.0	2.0	1.5	2.0	1.5	2.5
Chemical	0.0	0.0	0.0	0.0	0.0	0.0	0.0	2.0
Fat	3.0	3.5	4.0	4.0	4.0	4.0	4.5	4.0
Protein	3.0	3.0	3.0	4.0	1.0	2.0	2.0	3.5
Brown/Roasted	4.0	5.0	5.0	4.5	5.0	5.5	5.5	5.0
Warmed-Over Flavor	3.0	2.5	2.5	2.5	3.0	2.5	2.0	3.0
Rancid	0.0	0.0	0.0	0.0	0.0	2.0	0.0	0.0
Salty	2.5	3.5	3.0	3.5	3.5	3.5	3.5	3.0
Sour	1.5	2.5	1.5	2.0	2.5	2.0	2.5	2.0
Bitter	2.0	2.0	2.0	2.0	2.0	2.0	2.0	2.0
**Mouthfeel**								
Slick	0.0	0.0	0.0	0.0	0.0	0.0	2.0	0.0
Astringent	1.5	1.5	1.5	1.5	3.0	2.5	3.0	3.0
